# Supplementary material for: Assembling covalent organic framework membranes with superior ion exchange capacity
Source: Nat Commun. 2022 Feb 23;13:1020. doi: 10.1038/s41467-022-28643-8 (PMC8866435; doi:10.1038/s41467-022-28643-8)
Supplement: Supplementary file 1 — Supplementary Information [file 41467_2022_28643_MOESM1_ESM.pdf]

Supplementary Information

Assembling covalent organic framework membranes with superior ion exchange capacity

Xiaoyao Wang[1,2], Benbing Shi[1,2], Hao Yang[3], Jingyuan Guan[1,2], Xu Liang[1,2], Chunyang Fan[1,2], Xinda You[1,2], Yanan Wang[1,2], Zhe Zhang[3], Hong Wu[1,2,4], Tao Cheng[3], Runnan Zhang[1,2,6], Zhongyi Jiang[1,2,5,6*]*

1. Materials and methods

1.1 Fabrication of TpPa-SO_3H (NUS-9) iCOFMs

0.2 mmol (42.0 mg) of 2,4,6-triformylphloroglucinol (Tp) was dissolved into 20 mL n-octanoic acid by sonication for 30 min. 0.3 mmol (56.4 mg) of 2,5-diaminobenzenesulfonic acid (Pa-SO_3H) and 0.3 mmol (1 equivalent) of sodium formate was dissolved into 30 mL deionized water by sonication for 20 min. The amine monomer solution was poured into the bottom of 100 mL beaker, and the Tp solution was added by drops on the top layer. Under static conditions for 24 hours, the TpPa-SO_3H iCOFMs could be taken out from the beaker using tweezers. After washing with DMF and ethyl alcohol, TpPa-SO_3H iCOFMs was taken out for further characterization and performances evaluation.

1.2 Synthesis of TpPa-SO_3H (NUS-9) powders

Typically, 0.2 mmol of 2,4,6-triformylphloroglucinol (Tp), 0.3 mmol (56.4 mg) of 2,5-diaminobenzenesulfonic acid (Pa-SO_3H), 3 mL mesitylene, 1 mL dioxane and acetic acid (6 mol L^{-1}, 0.5 mL) were added in a 15 mL pyrex tube. The mixture was sonicated for 20 min and frozen in liquid nitrogen, followed by degassing treatment by three freeze-pump-thaw cycles. Then, the tube was sealed and heated at 120 °C for 3 days. The red powder was collected by filtration, washed with anhydrous ethanol, anhydrous acetone and deionized water for 3 times and subjected to Soxhlet extraction with methanol for 3 days. Finally, the red powder was collected after drying at 120 °C under vacuum. Yield: 70.6 mg, 71.8%.

1.3 IEC determination

The IEC values of the iCOFMs were measured by a titration method. The dry samples (0.2 g) were added into NaCl solution (0.01 g/mL, 20 mL) and stirred for 24 h. Then the solutions were titrated using a 0.01 M NaOH standard solution, and phenolphthalein was used as indicator. The IEC values (mmol g^{-1}) of the samples were calculated by Supplementary Equation 1:

$$IEC = \frac{C_{NaOH} \times V_{NaOH}}{W_{Dry}} \times 100\% \quad (1)$$

where C_{NaOH} (mol L^{-1}) is the molar concentration of the NaOH solution; V_{NaOH} (L) is the consumed volume of the NaOH solution during the titration process; and W_{Dry} (g) is the weight of the dry samples.

2. COF structure modelling and simulation

The predicted structures of crystalline TpBD-$(SO_3H)_2$ iCOFMs were carried out using Materials Studio (MS) software. AA stacking model and reversed AA stacking model were simulated to elucidate the structures of TpBD-$(SO_3H)_2$ iCOFMs. The initial lattice was built by starting with the space group P1, inserted the optimized monomer in the empty cell, omitted the redundant atoms, and promoted the symmetry to P6/M, producing the crude COF structures. Then, MS Forcite molecular dynamics module was used to geometry-optimize the lattice model. Simulated PXRD pattern was done using Reflex module of MS. The fractional coordinates of TpBD-$(SO_3H)_2$ iCOFMs are shown in Supplementary Figure 5 and Supplementary Table 1.

3. Supplementary Discussions

3.1 Discussion on the crystallinity of TpBD-$(SO_3H)_2$ COF powder and TpBD-$(SO_3H)_2$ iCOFMs

Previous studies have proved that suitable reaction rate and reversibility and low growth rate are reliable conditions for the synthesis of high crystallinity COFs[1-3]. On one hand, the solvothermal reaction system for COF powder synthesis was homogeneous. We can only activate one type of monomer by adding either aldehyde monomer activator (acid) or amine monomer activator (base). On the other hand, during the homogeneous solvothermal synthesis for COF powder, the two monomers directly contact and polymerize rapidly at high temperatures. During the interfacial polymerization process for the fabrication of iCOFMs, the growth rate was limited by the diffusion rate of the monomers across the interface, which can maintain efficient self-correction for higher crystallinity.

3.2 Discussion on the crystallinity of the top dense side and the loosely stacked side of TpBD-$(SO_3H)_2$ iCOFMs

As shown in Supplementary Figure 5, it was found that the crystallinity of the top dense side of the membrane was slightly higher than that of the loosely stacked side. This is because the crystallinity of COFs was significantly affected by the growth rate[1]. In this study, when the interfacial polymerization started, the two-phase monomers reacted rapidly, and the faster growth rate could result in COF particles with relatively low crystallinity[2]. As the reaction time prolonged, the COF particles will stack into a loose layer, which reduced the further diffusion rate of amine monomers into the organic phase. This is similar to the self-sealing and self-terminating behavior in the interfacial polymerization process for polyamide membranes[3,4]. Accordingly, the interfacial polymerization was slowed down, leading to the formation of a compact layer on top side. The slower growth rate can maintain efficient self-correction of defects, leading to higher crystallinity of the top dense layer[2,5].

3.3 Discussion on the selection criteria of activators

As shown in Supplementary Figure 12, we cannot obtain membranes when using a stronger acid (acetic acid) as the aldehyde monomer activator. This was because the acetic acid may diffuse into water and the base cannot sufficiently activate the amine monomer in the water phase. Moreover, we cannot obtain membranes if the amine monomer activator was a strong base (sodium hydroxide), since the strong base would immediately neutralize the acid at the interface. Therefore, we can obtain the selection criteria of the activators. The aldehyde monomer activator needs to be immiscible with water to form a stable interface and avoid diffusing into water phase. The basicity of the amine monomer activator needs to be strong enough to extract protons from the ionic amine monomer, but should not be too strong to avoid neutralization with the acid in the organic phase. The addition amount as well as the acidity (basicity) of the activator can be adjusted according to the monomer reactivity as well as required membrane thickness and crystallinity."

4. Figures and tables

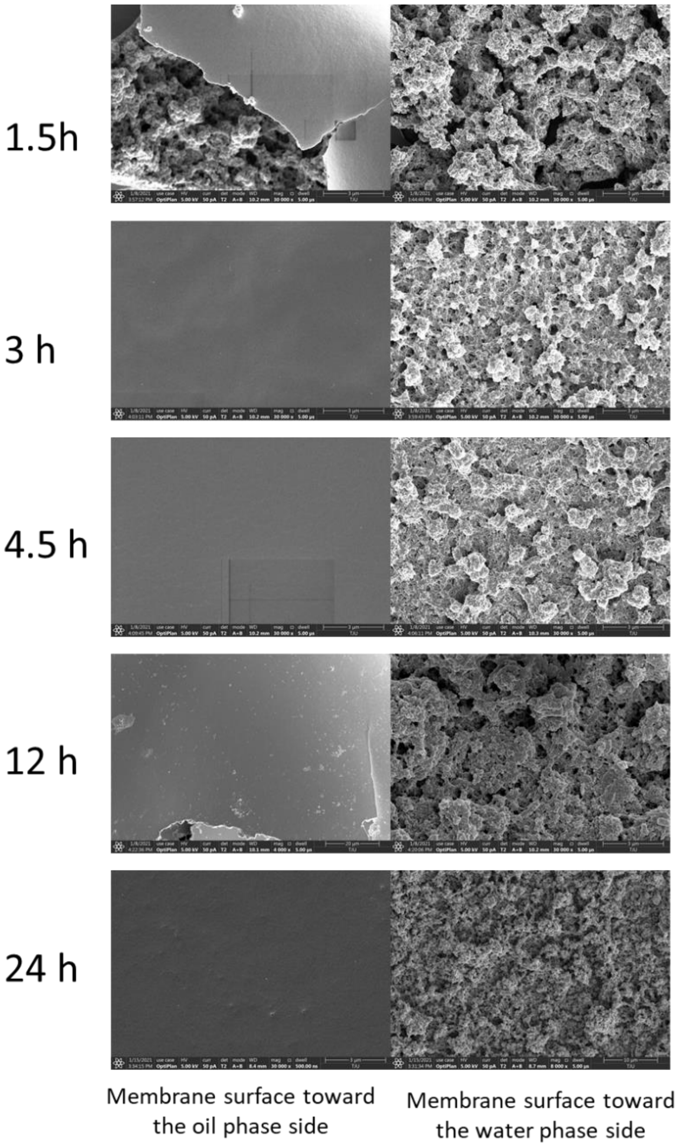

Supplementary Figure 1. SEM images of TpBD-$(SO_3H)_2$ iCOFMs at different times (1.5-24h).

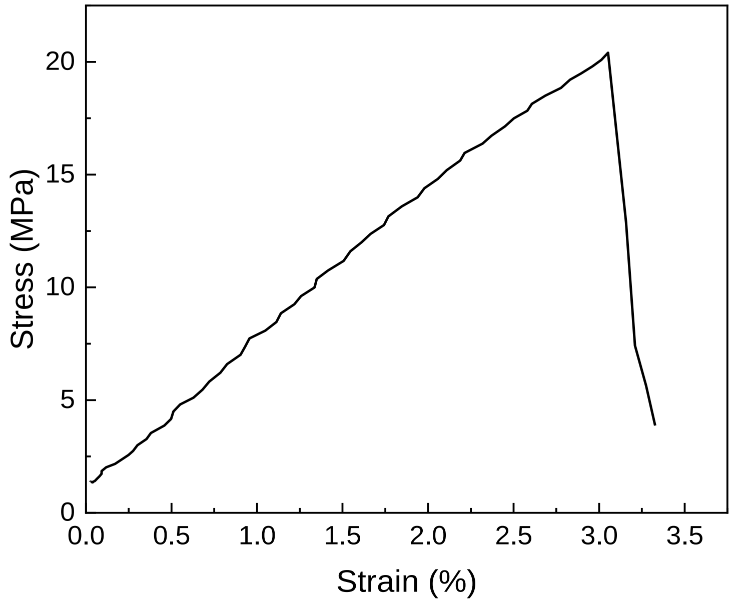

Supplementary Figure 2. mechanical strength of TpBD-$(SO_3H)_2$ iCOFMs.

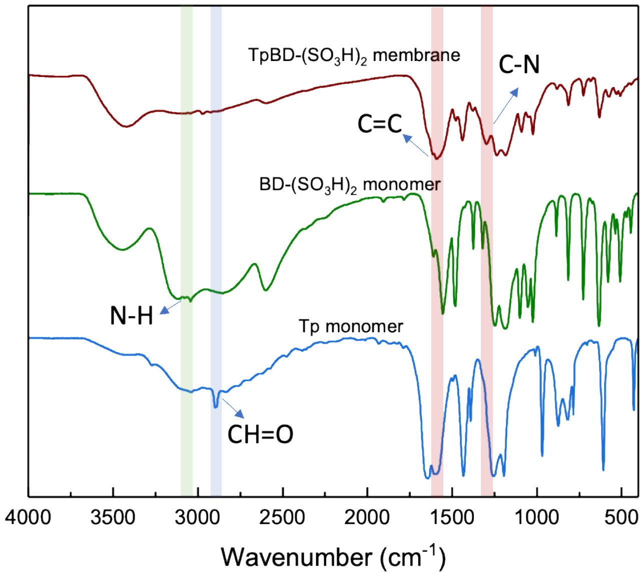

Supplementary Figure 3. FTIR spectra of TpBD-$(SO_3H)_2$ iCOFMs.

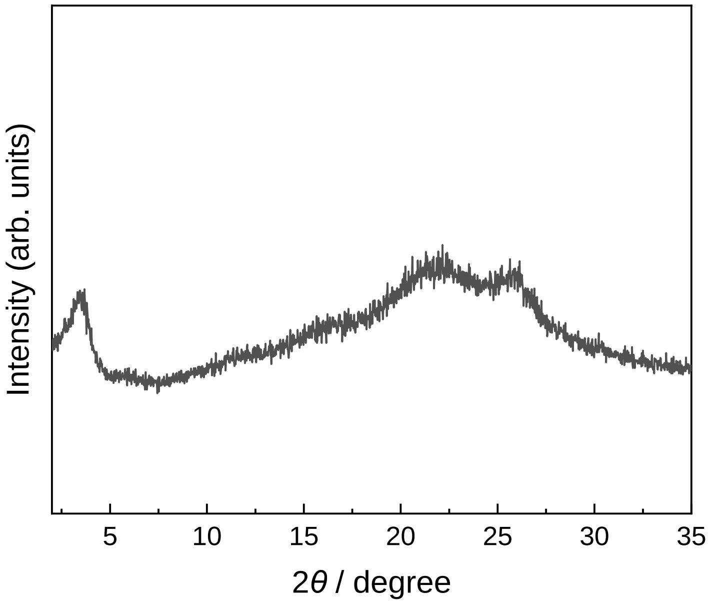

Supplementary Figure 4. PXRD patterns of the TpBD-$(SO_3H)_2$ powder sample synthesized by solvothermal method.

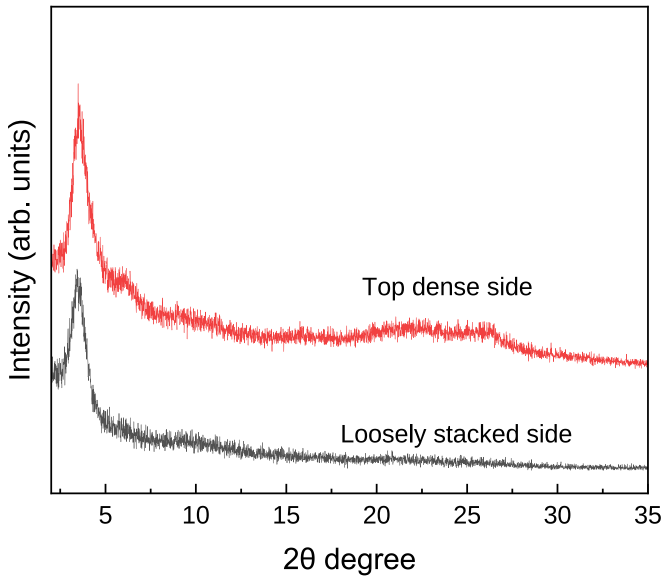

Supplementary Figure 5. GIXRD patten of top dense side and loosely stacked side of the TpBD-$(SO_3H)_2$ iCOFMs.

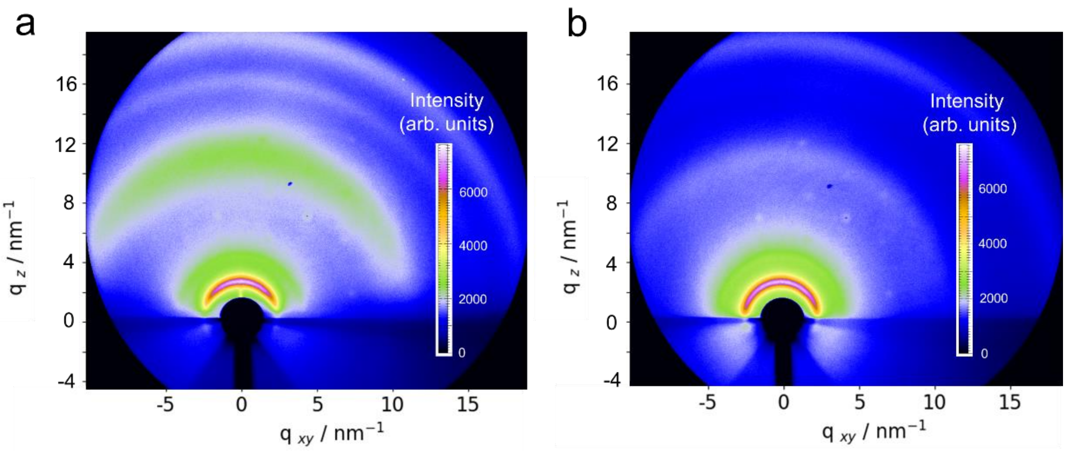

Supplementary Figure 6. GIWAXS patten of (a) top dense side and (b) loosely stacked side of the TpBD-$(SO_3H)_2$ iCOFMs.

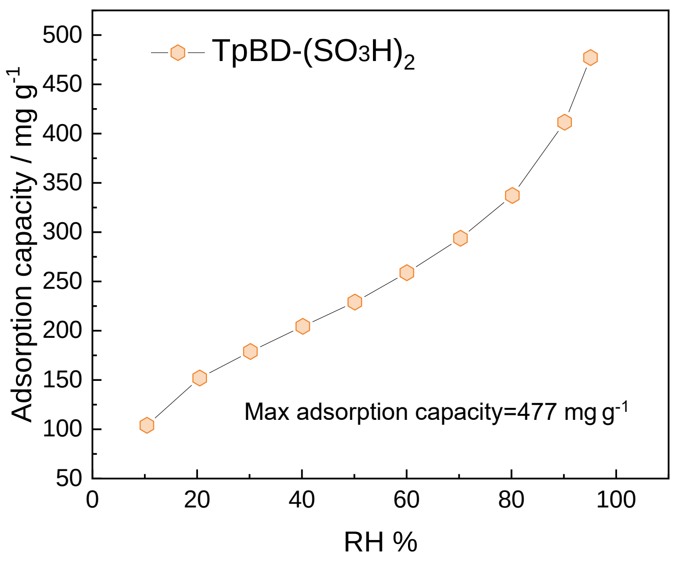

Supplementary Figure 7. Vacuum water vapor sorption isotherm of TpBD-(SO_3H)$_2$ iCOFMs.

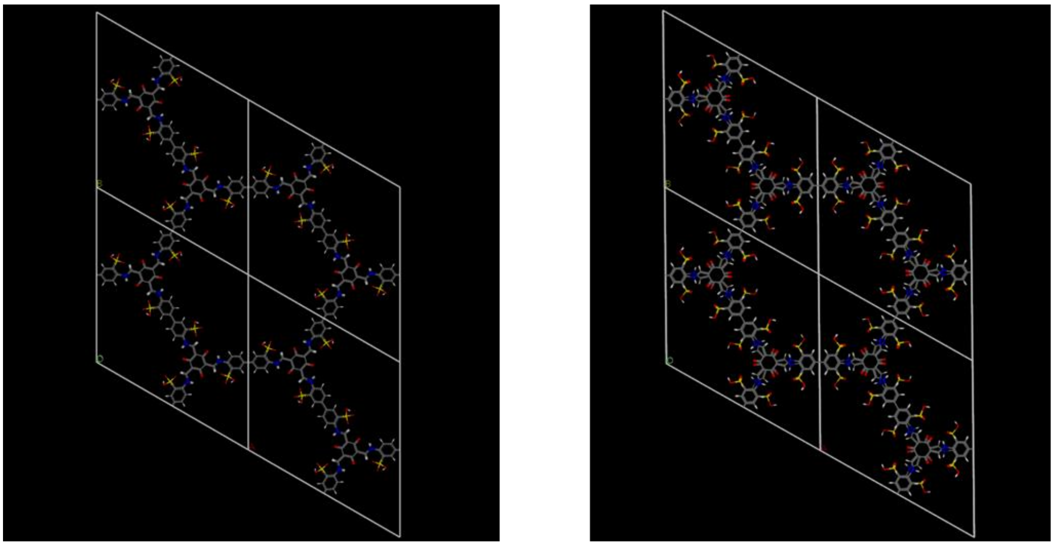

AA stacking model Reversed AA stacking model

Supplementary Figure 8. AA stacking model and reversed AA stacking model simulated by Materials Studio (MS) program.

Supplementary Table 1. Detailed atom information of the simulated TpBD-$(SO_3H)_2$ COF in reversed AA Stacking

a= 31.2447, b=31.2447, c=6.84684; alpha=beta=90°, gamma = 120°; P6/M space group

O1	-3.04	22.455	4.63
O2	13.52	7.351	3.423
C3	14.478	8.094	3.423
C4	14.212	9.561	3.423
C5	12.898	9.966	3.423
H6	17.206	5.959	3.423
C7	16.716	2.957	3.423
C8	16.745	1.553	3.423
C9	15.583	0.759	3.423
C10	14.354	1.448	3.423
C11	14.3	2.843	3.423
C12	15.457	3.644	3.423
H13	17.721	1.107	3.423
H14	13.399	0.948	3.423
H15	13.32	3.313	3.423
N16	0.402	21.974	3.423
H17	14.21	5.326	3.423
S18	-2.763	23.379	3.423
O19	-4.104	24.463	3.423
H20	-4.887	23.851	3.423

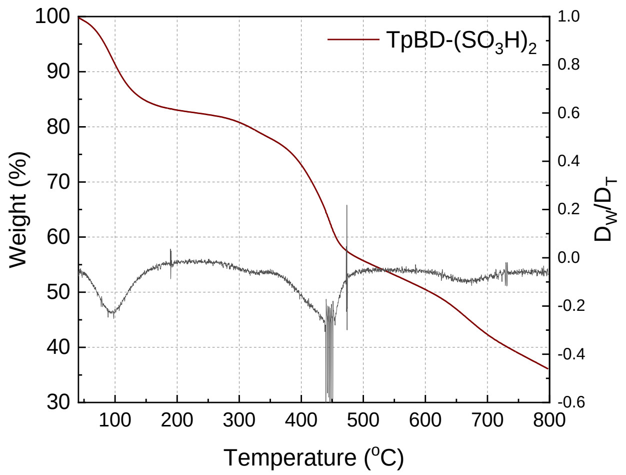

Supplementary Figure 9. TGA curve of TpBD-$(SO_3H)_2$ membrane from 40 to 800 ºC at a heating rate of 10 ºC min^{-1} under N_2. The membrane shows two weight loss. The first weight loss (from 40-100 ºC) is related to the loss of absorbed water in the hydrophilic channels. The second weight loss in the range of 350-800 ºC is from the disintegration of sulfonic acid groups the decomposition of membrane backbones.

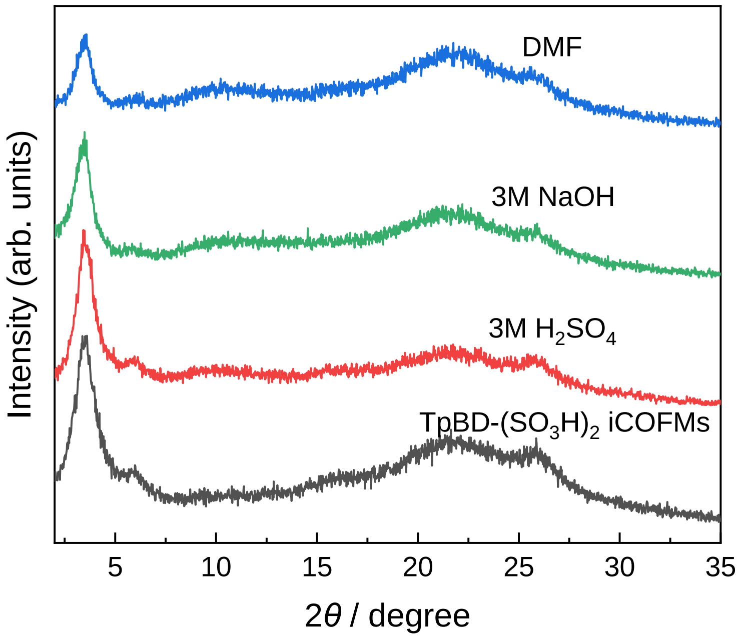

Supplementary Figure 10. PXRD patterns of the TpBD-$(SO_3H)_2$ iCOFMs samples after immersing in 3M H_2SO_4 and 3M NaOH at room temperature and N, N-dimethylformamide at 100 ºC for 5 days.

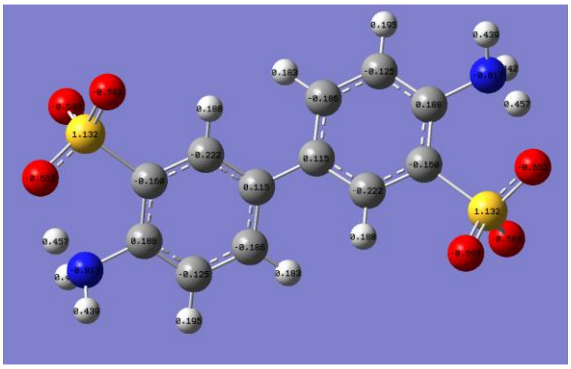

Supplementary Figure 11. Optimized structure and Mulliken charges of BD-$(SO_3H)_2$ monomers.

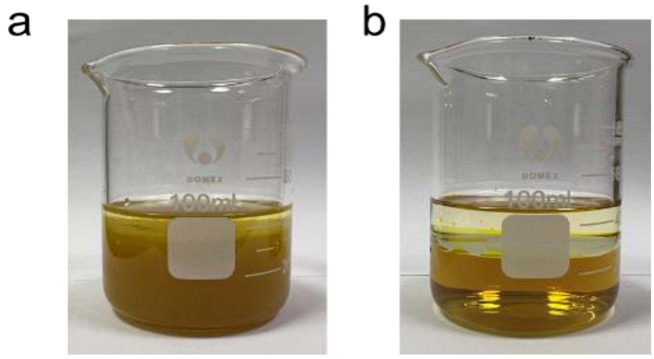

Supplementary Figure 12. Fabrication of the iCOFMs using IP technology with (a) stronger acid (acetic acid) and (b) strong base (sodium hydroxide).

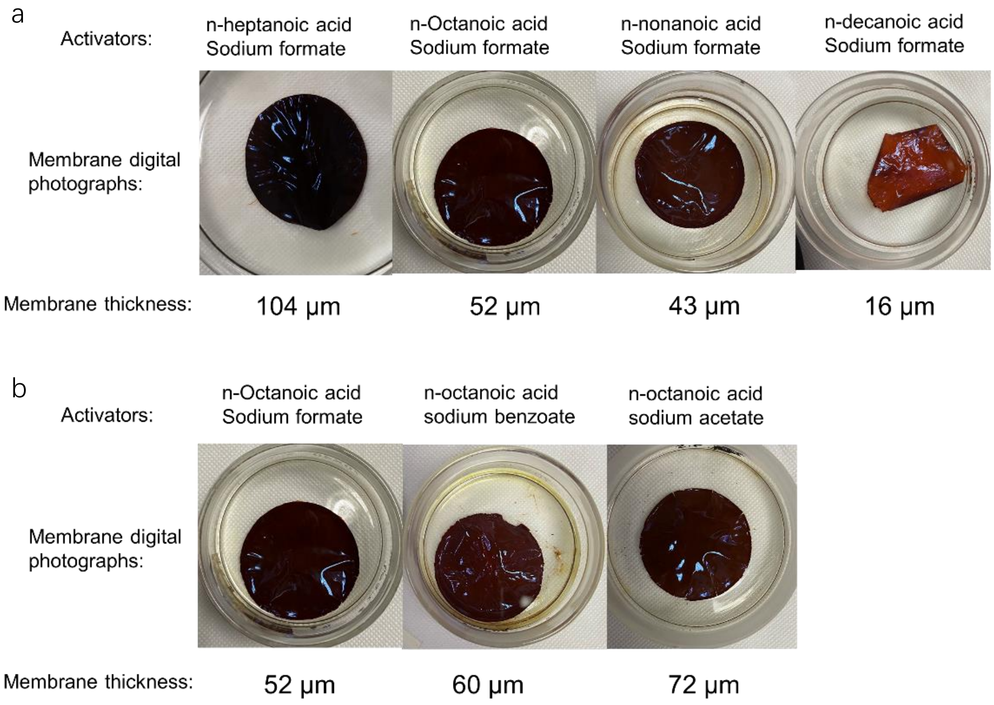

Supplementary Figure 13. Digital photos of TpBD-$(SO_3H)_2$ iCOFMs (Fabrication condition: a. 15 mL n-octanoic acid for aldehyde monomer activation and 2.0 eq sodium formate/ sodium acetate/ sodium benzoate for amine monomer activation; b. 15 mL n-heptanoic acid/ n-octanoic acid/ n-nonanoic acid/ n-decanoic acid for aldehyde monomer activation and 2.0 eq sodium acetate for amine monomer activation).

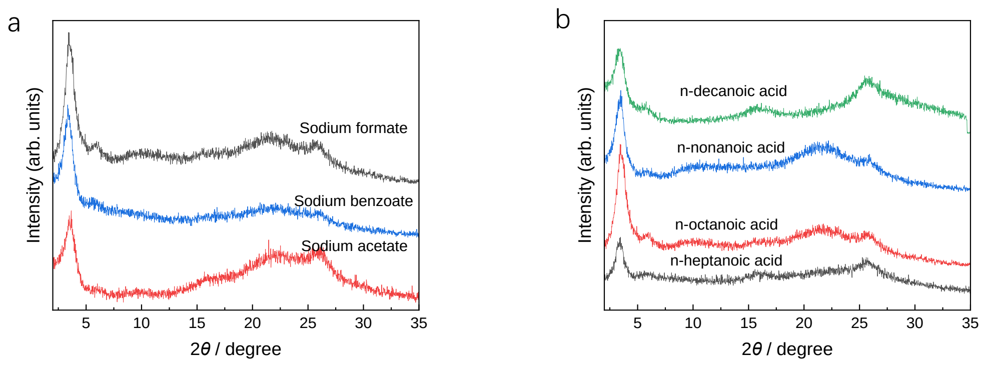

Supplementary Figure 14. PXRD patterns of TpBD-$(SO_3H)_2$ iCOFMs (Fabrication condition: a. 15 mL n-octanoic acid for aldehyde monomer activation and 2.0 eq sodium formate/ sodium acetate/ sodium benzoate for amine monomer activation; b. 15 mL n-heptanoic acid/ n-octanoic acid/ n-nonanoic acid/ n-decanoic acid for aldehyde monomer activation and 2.0 eq sodium acetate for amine monomer activation).

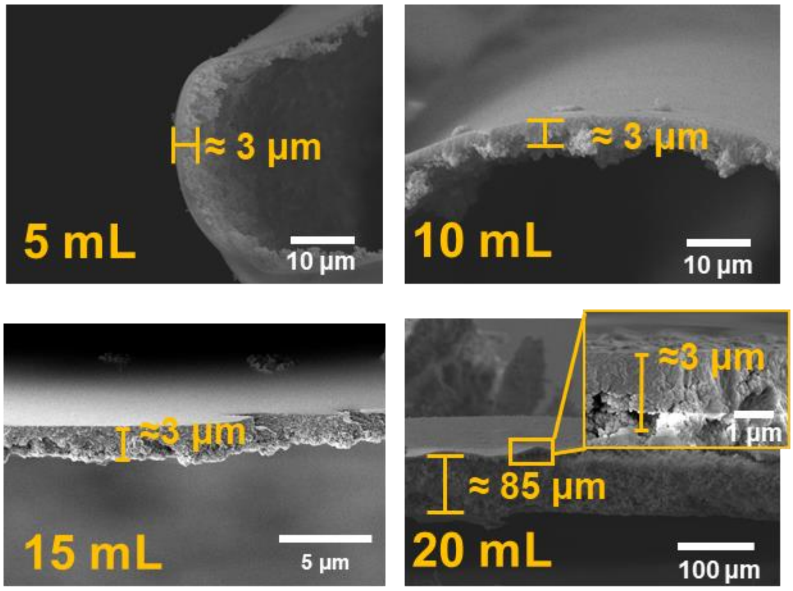

Supplementary Figure 15. Effect of the addition amount of aldehyde monomer activator on the thickness of TpBD-$(SO_3H)_2$ iCOFMs.

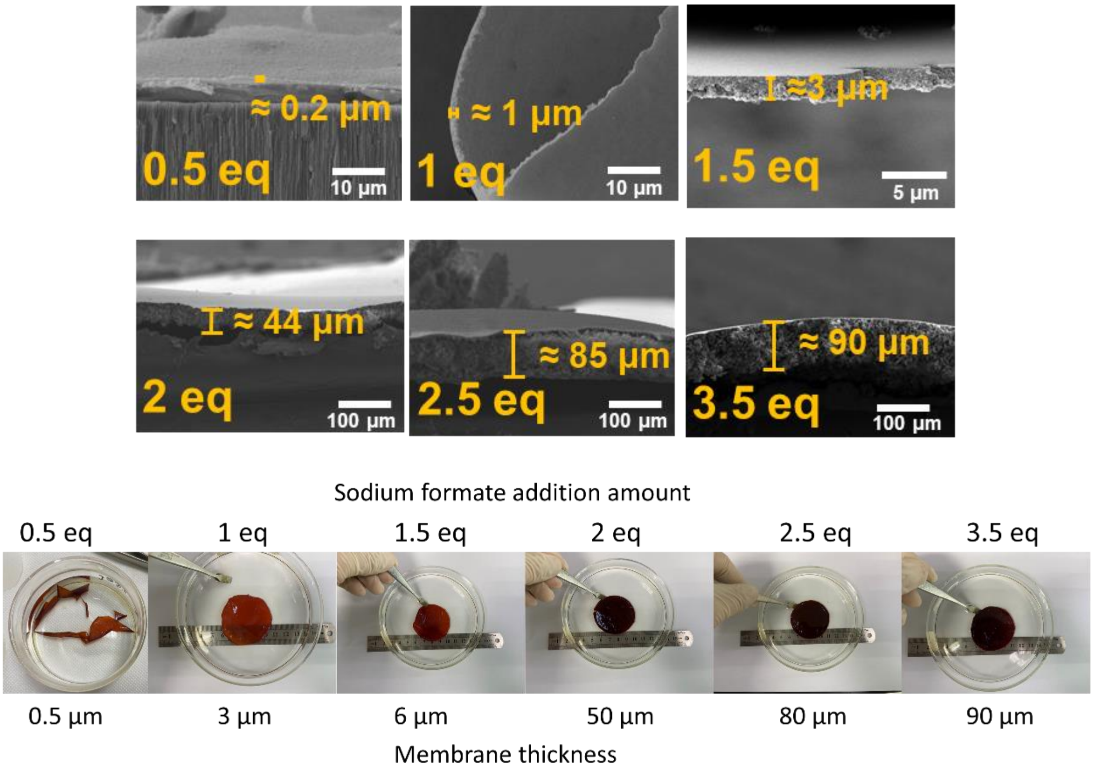

Supplementary Figure 16. Effect of the addition amount of amine monomer activator on the thickness of TpBD-$(SO_3H)_2$ iCOFMs.

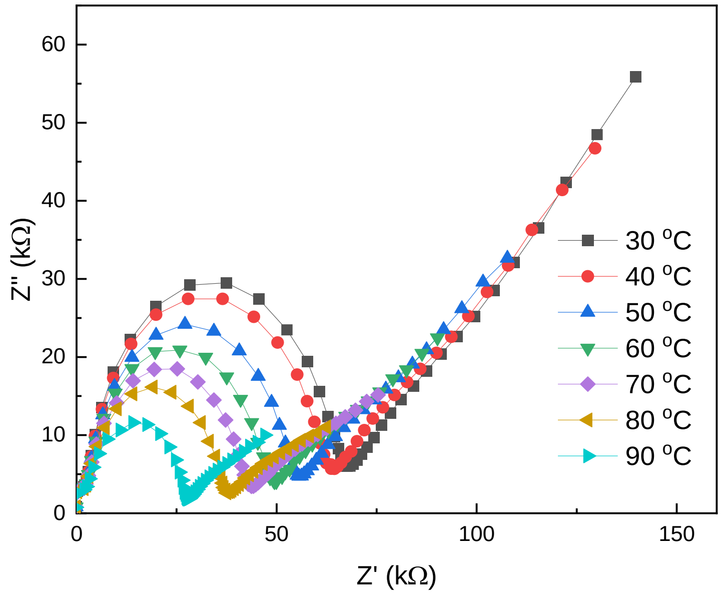

Supplementary Figure 17. Nyquist plots of TpBD-$(SO_3H)_2$ iCOFMs under 100% RH at elevated temperature.

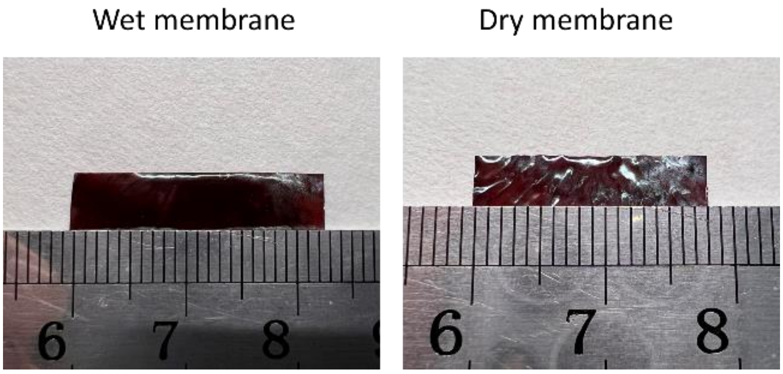

Supplementary Figure 18. Digital photographs of wet and dry TpBD-$(SO_3H)_2$ iCOFMs.

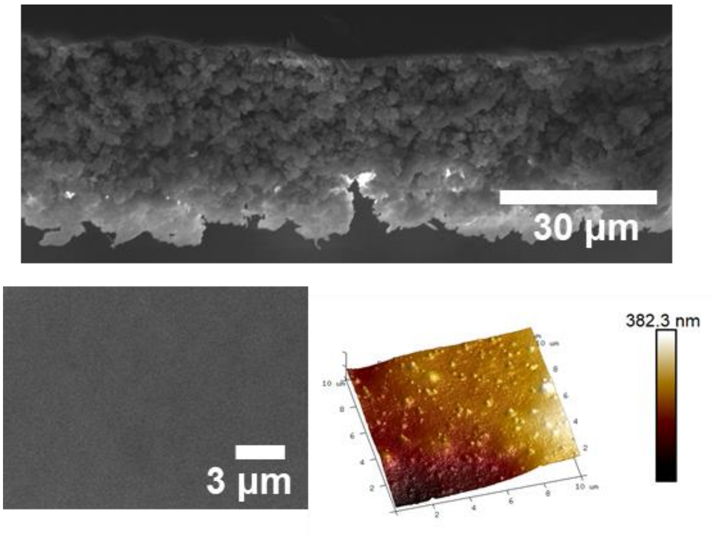

Supplementary Figure 19. Cross-section SEM image, surface SEM image and surface AFM images of TpPa-SO_3H iCOFMs.

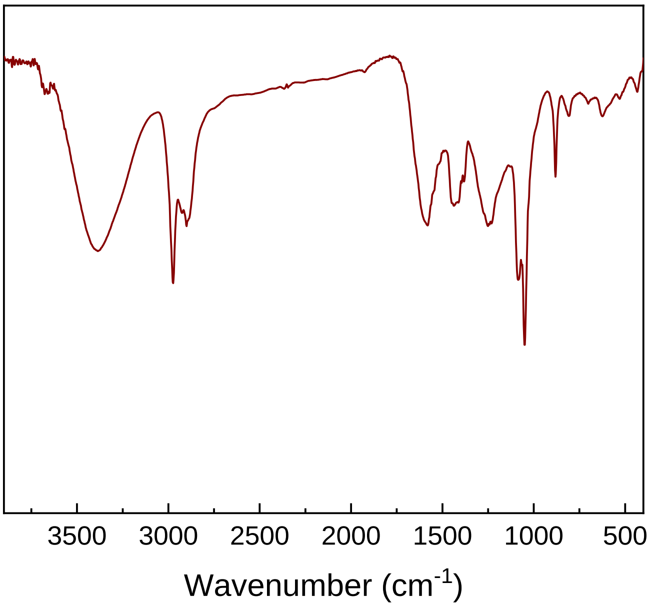

Supplementary Figure 20. FTIR spectra of TpPa-SO_3H iCOFMs.

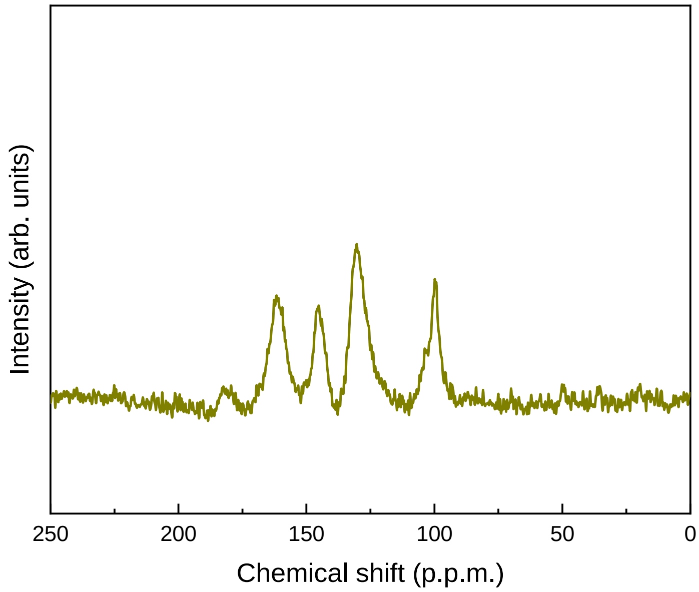

Supplementary Figure 21. Solid-state ^{13}C NMR spectra of TpPa-SO_3H iCOFMs.

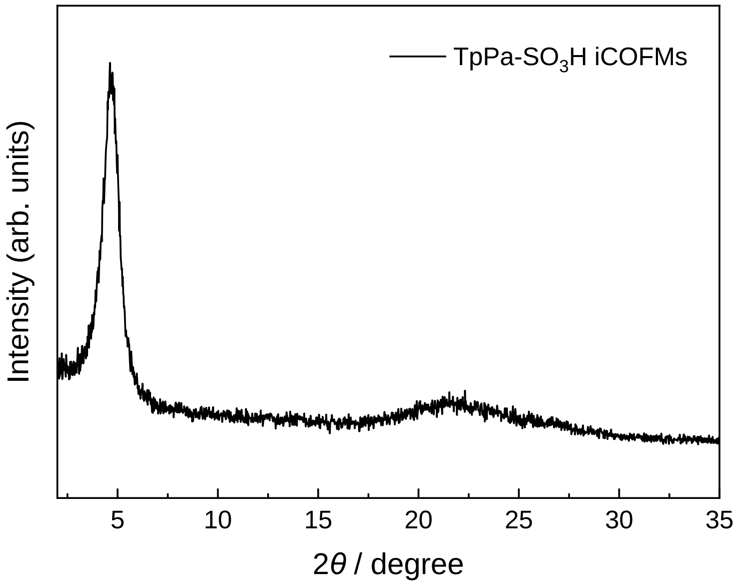

Supplementary Figure 22. PXRD patterns of TpPa-SO_3H iCOFMs.

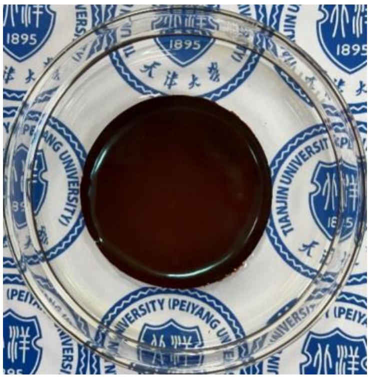

Supplementary Figure 23. Digital photo of TpPa-SO_3H iCOFMs.

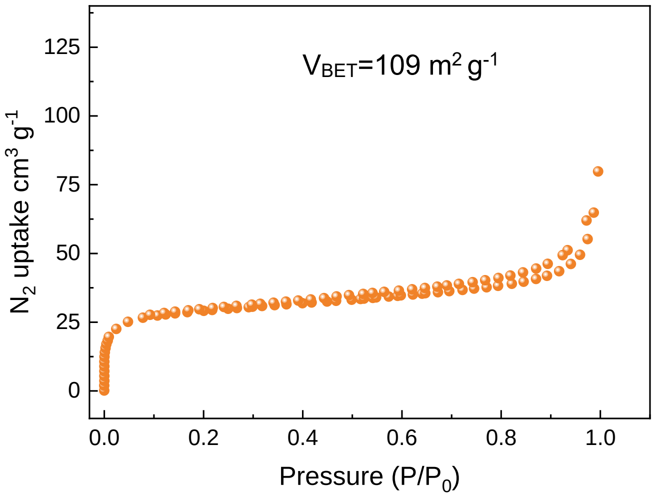

Supplementary Figure 24. N_2 sorption isotherms of TpPa-SO_3H iCOFMs.

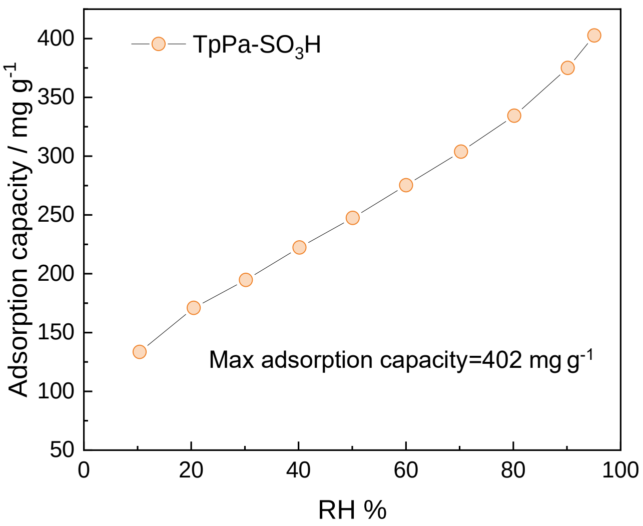

Supplementary Figure 25. Vacuum water vapor sorption isotherm of TpPa-SO_3H iCOFMs.

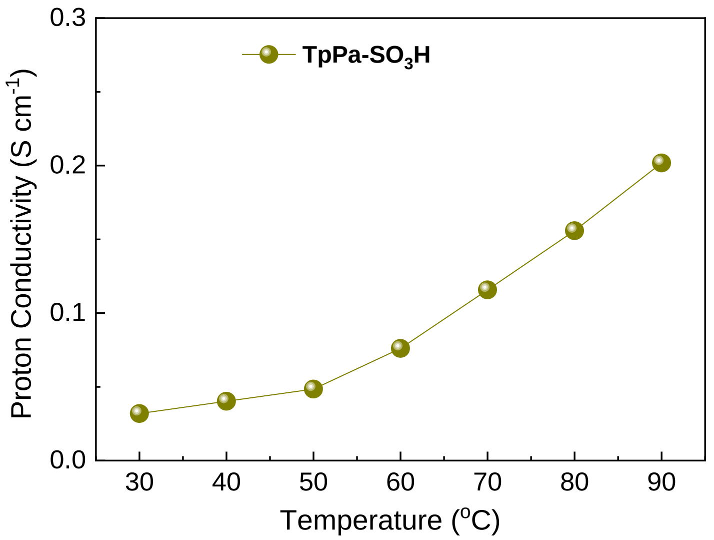

Supplementary Figure 26. Proton conductivity of TpPa-SO_3H iCOFMs.

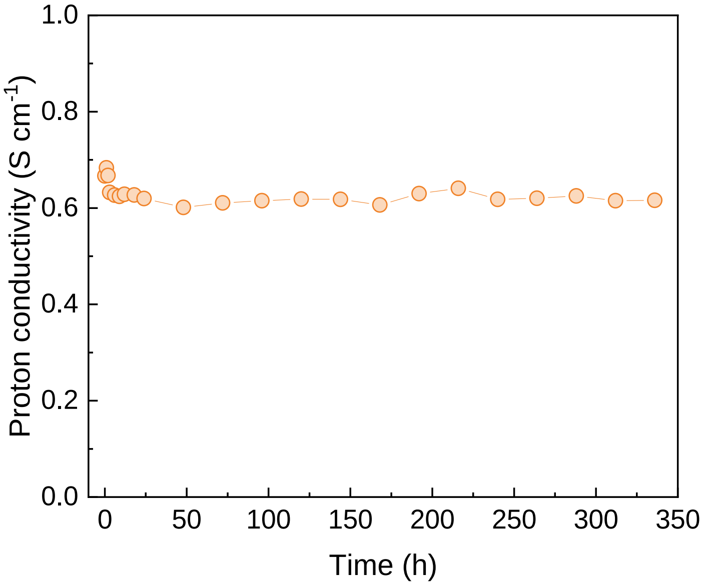

Supplementary Figure 27. long-term proton conductivity of the TpBD-$(SO_3H)_2$ iCOFMs.

Supplementary Table 2. Fukui function for electrophilic attack sites (f^-) of non-ionic amine monomers

Amine monomers	Fukui function for Electrophilic Attack f^-	
p-Phenylenediamine 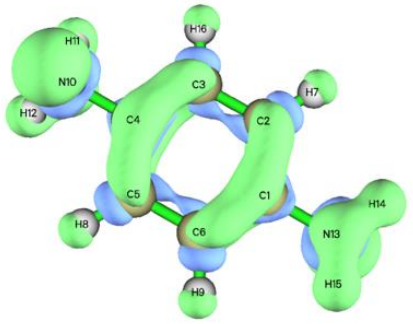	1(C）	0.0764
	2(C)	0.0636
	3(C)	0.0636
	4(C)	0.0764
	5(C)	0.0636
	6(C)	0.0636
	7(H)	0.039
	8(H)	0.039
	9(H)	0.039
	10(N)	0.1237
	11(H)	0.0474
	12(H)	0.0474
	13(N)	0.1237
	14(H)	0.0474
	15(H)	0.0474
	16(H)	0.039
Benzidine 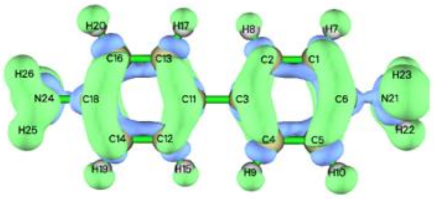	1(C)	0.0439
	2(C)	0.0358
	3(C)	0.0433
	4(C)	0.0358
	5(C)	0.0438
	6(C)	0.0505
	7(H)	0.0277
	8(H)	0.0221
	9(H)	0.0221
	10(H)	0.0277
	11(C)	0.0432
	12(C)	0.0358
	13(C)	0.0358
	14(C)	0.0437
	15(H)	0.022
	16(C)	0.0438
	17(H)	0.0221
	18(C)	0.0505
	19(H)	0.0277
	20(H)	0.0277
	21(N)	0.0822
	22(H)	0.0328
	23(H)	0.0326
	24(N)	0.0821
	25(H)	0.0328

	26(H)	0.0326

Supplementary Table 3. Fukui function for electrophilic attack sites (f^-) of ionic amine monomers

Amine monomers	Fukui function for Electrophilic Attack f^-	
	1(C)	0.0361
	2(C)	0.0346
	3(C)	0.0065
2,5-Diaminobenzenesulfonic acid	4(C)	0.0463
	5(C)	0.036
	6(C)	0.0536
	7(H)	0.0172
	8(H)	0.0284
	9(H)	0.0302
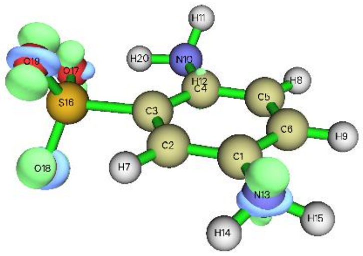	10(N)	0.0172
	11(H)	0.0216
	12(H)	0.0217
	13(N)	0.0924
	14(H)	0.032
	15(H)	0.0358
	16(S)	0.0653
	17(O)	0.1068
	18(O)	0.1522
	19(O)	0.1536
	20(H)	0.0124
	1(C)	0.0171
	2(C)	0.0162
	3(C)	0.0067
4,4'-diaminobiphenyl-3,3'-disulphonic acid ($BD-(SO_3H)_2$)	4(C)	0.004
	5(C)	-0.0023
	6(C)	0.0096
	7(H)	0.0146
	8(H)	0.0084
	9(H)	0.004
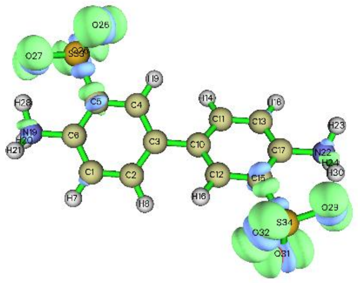	10(C)	0.0067
	11(C)	0.0162
	12(C)	0.004
	13(C)	0.0171
	14(H)	0.0084
	15(C)	-0.0022
	16(H)	0.004
	17(C)	0.0097
	18(H)	0.0146
	19(N)	0.0089
	20(H)	0.0107
	21(H)	0.0126

	22(N)	0.0089
	23(H)	0.0126
	24(H)	0.0107
	25(O)	0.1202
	26(O)	0.1162
	27(O)	0.0919
	28(H)	0.0085
	29(O)	0.0919
	30(H)	0.0085
	31(O)	0.1201
	32(O)	0.1162
	33(S)	0.0527
	34(S)	0.0526

Supplementary Table 4. Fukui function for electrophilic attack sites (f^-) of ionic amine monomers after activation

Amine monomers	Fukui function for Electrophilic Attack f^-	
2,5-Diaminobenzenesulfonic acid	1(C)	0.063
	2(C)	0.0351
	3(C)	0.0321
	4(C)	0.0497
	5(C)	0.0542
	6(C)	0.0544
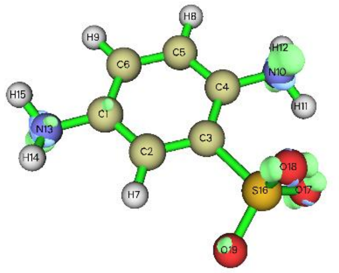	7(H)	0.0241
	8(H)	0.0338
	9(H)	0.0348
	10(N)	0.1034
	11(H)	0.0291
	12(H)	0.0429
	13(N)	0.0801
	14(H)	0.0342
	15(H)	0.036
	16(S)	0.0373
	17(O)	0.0843
	18(O)	0.0943
	19(O)	0.0774
4,4'-diaminobiphenyl-3,3'-disulphonic acid ($BD-(SO_3H)_2$)	1(C)	0.0376
	2(C)	0.0327
	3(C)	0.0366
	4(C)	0.0226
	5(C)	0.0269
	6(C)	0.0407
	7(H)	0.0245
	8(H)	0.0191
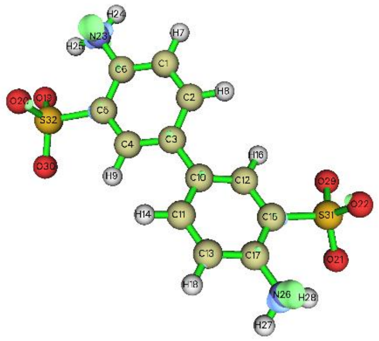	9(H)	0.0143
	10(C)	0.0367
	11(C)	0.0327
	12(C)	0.0226
	13(C)	0.0376
	14(H)	0.0192
	15(C)	0.027
	16(H)	0.0143
	17(C)	0.0408
	18(H)	0.0246
	19(O)	0.0347
	20(O)	0.0414
	21(O)	0.0347
	22(O)	0.0414

	23(N)	0.0719
	24(H)	0.03
	25(H)	0.0216
	26(N)	0.072
	27(H)	0.03
	28(H)	0.0216
	29(O)	0.0305
	30(O)	0.0305
	31(S)	0.0146
	H (32)	0.025

Supplementary Table 5. Fukui function for nucleophilic attack sites(f^+) of aldehyde monomers

Aldehyde monomers	Fukui function for nucleophilic attack (f^+)	
2,4,6-triformylphloroglucinol (Tp) 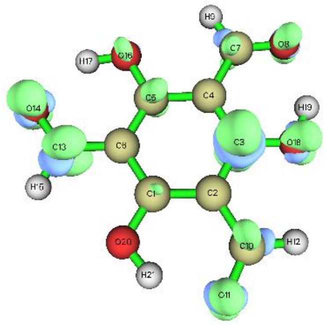	1(C)	0.0284
	2(C)	0.0249
	3(C)	0.1055
	4(C)	0.0246
	5(C)	0.0427
	6(C)	0.0264
	7(C)	0.0483
	8(O)	0.0638
	9(H)	0.0302
	10(C)	0.0597
	11(O)	0.0785
	12(H)	0.0297
	13(C)	0.0908
	14(O)	0.0952
	15(H)	0.0396
	16(O)	0.046
	17(H)	0.0186
	18(O)	0.0721
	19(H)	0.0232
	20(O)	0.0353
	21(H)	0.0166
	Average value of chemical equivalent reaction sites:	0.0662

Supplementary Table 6. Fukui function for nucleophilic attack sites(f^{+}) of aldehyde monomers after activation

Aldehyde monomers	Fukui function for nucleophilic attack (f^{+})	
	1(C)	0.0549
	2(C)	0.0276
	3(C)	0.0606
	4(C)	0.0368
2,4,6-triformylphloroglucinol (Tp)	5(C)	0.0421
	6(C)	0.0226
	7(C)	0.1541
	8(H)	0.054
	9(C)	0.0303
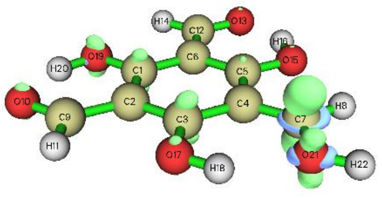	10(O)	0.0573
	11(H)	0.0223
	12(C)	0.027
	13(O)	0.05
	14(H)	0.0225
	15(O)	0.0416
	16(H)	0.0175
	17(O)	0.057
	18(H)	0.0184
	19(O)	0.0642
	20(H)	0.0186
	21(O)	0.0806
	22(H)	0.0399

Supplementary Table 7. Swelling ratio and water uptake of TpBD-$(SO_3H)_2$ iCOFMs and as reported in literature.

Membranes	IEC (mmol g^{-1})	Swelling ratio (%)		Temperature (°C)	Conditions	Water uptake (%)	Ref.
		Thickness	Area				
TpBD-$(SO_3H)_2$	4.6	22	21	25	DI water	144.4	This work
TpBD-$(SO_3H)_2$		25	23	80			
TpPa-SO_3H	3.2	11	14	25		75.3	This work
TpPa-SO_3H		15	16	80			
IPC-COF membrane	3.2	9	1	25		60	[6]
Random copolymer	3.28	110	33	80		183.3	[7]
	3.19	77	38	80		119	[7]
	3.26	160	132	50		44	[8]
	2.51	95	180	50		35	[8]
Block polymer	1.83	70	25	20		91.2	[9]
	1.50	65	11	30		48	[10]
	1.67	55	16	25		82	[11]
	1.83	10		20		25	[12]
Nafion 212®	0.93	14	14	25		12	[6]
	0.93	21	44	80		36	

Supplementary Table 8. Compared proton conductivity for previously reported COF materials and this work

Sr. No	System	Proton conductivities (S cm^{-1})	Measurement conditions	Ref.
1	TpBD-$(SO_3H)_2$	6.6×10^{-1}	90 °C, 100% RH	This work
2	TpPa-SO_3H	2×10^{-1}	90 °C, 100% RH	This work
2	Phytic@TpPa-(SO_3H-Py)	5×10^{-4}	120 °C, anhydrous	[13]
3	PA@TpBpy-MC	2.5×10^{-3}	393 K, anhydrous	[14]
4	RT-COF-1AcB	5.25×10^{-4}	313 K, 100% RH	[15]
5	EBCOF: PW_{12}	3.32×10^{-3}	25 °C, 97% RH	[16]
6	im@TPB-DMTP-COF	4.37×10^{-3}	120 °C, anhydrous	[17]
7	PA@Tp-Azo	9.9×10^{-4}	332 K, 98% RH	[18]
8	PTSA@TpAzo	7.8×10^{-2}	80 °C, 95% RH	[19]
9	NUS-10(R)	3.96×10^{-2}	298 K, 97% RH	[20]
10	H_3PO_4@NKCOF-1	1.13×10^{-1}	353K, 98% RH	[21]
11	H_3PO_4@TPB-DMeTP-COF	1.91×10^{-1}	160 °C, anhydrous	[22]
12	Im@Py-TT-COF-50	3.08×10^{-3}	130 °C, anhydrous	[23]
13	BIP	3.2×10^{-2}	95 °C, 95% RH	[24]
14	aza-COF-2_H	4.80×10^{-3}	323 K, 97% RH	[25]
15	PA@EB-COF	2.77×10^{-2}	180 °C, anhydrous	[26]
16	COF-F6-H	4.2×10^{-2}	140 °C, anhydrous	[27]
17	IPC-COF	3.8×10^{-1}	80 °C, 100% RH	[6]
18	SCOF	5.4×10^{-1}	80 °C, Pure water	[28]
19	HPW@TAPT-DHTA	5.3×10^{-1}	80 °C, 100% RH	[29]

Supplementary Table 9. Compared proton conductivity and IEC for previously reported state-of-the-art proton conducting membranes and this work

Sr. No	Membranes	Proton conductivities ($S\ cm^{-1}$)	Measurement conditions	IEC ($mmol\ g^{-1}$)	Ref.
1	$TpBD\text{-}(SO_3H)_2$	6.6×10^{-1}	90 °C, 100% RH	4.6	This work
2	$TpPa\text{-}SO_3H$	2×10^{-1}	90 °C, 100% RH	3.2	This work
3	$sPPN\text{-}H^+$	2.68×10^{-1}	80 °C, 95% RH	3.28	[7]
4	$sPPB\text{-}H^+$	1.72×10^{-1}	80 °C, 95% RH	3.19	[7]
5	Ionomer membrane	3×10^{-1}	80 °C, 93% RH	3.26	[8]
6	SP3O-b-PAES-b-SP3O	1.9×10^{-1}	20 °C in water	1.83	[9]
7	SP100	2.6×10^{-1}	120 °C, 100% RH	1.83	[12]
8	SPSN	1.6×10^{-1}	80 °C, 90% RH	1.56	[30]
9	SPP-QP	2.2×10^{-1}	80 °C, 95% RH	2.4	[31]
10	Sulfonated Block Copoly(ether ketone)s	2.9×10^{-1}	80 °C, 95% RH	1.8	[32]
11	SPAF-QP	2.9×10^{-1}	80 °C, 95% RH	1.97	[33]
12	P-PPSU	2.6×10^{-2}	80 °C,fully hydrated	2.75	[34]
13	IPC-COF	3.8×10^{-1}	80 °C, 100% RH	3.2	[6]
14	SCOF	5.4×10^{-1}	80 °C, Pure water	3.2	[28]
15	Nafion 212	7.5×10^{-2}	80 °C, 98% RH	0.93	[6]

Supplementary References

1 Ma, T. *et al.* Single-crystal x-ray diffraction structures of covalent organic frameworks. *Science* **361**, 48-52 (2018).

2 Liu, J. *et al.* Self-standing and flexible covalent organic framework (COF) membranes for molecular separation. *Sci. Adv.* **6**, eabb1110, doi:10.1126/sciadv.abb1110 (2020).

3 Li, Y. *et al.* Graphene quantum dot engineered ultrathin loose polyamide nanofilms for high-performance nanofiltration. *J. Mater. Chem. A* **8**, 23930-23938, doi:10.1039/D0TA09319J (2020).

4 Yuan, J. *et al.* Covalent organic framework-modulated interfacial polymerization for ultrathin desalination membranes. *J. Mater. Chem. A* **7**, 25641-25649, doi:10.1039/C9TA08163A (2019).

5 Karak, S., Kumar, S., Pachfule, P. & Banerjee, R. Porosity Prediction through Hydrogen Bonding in Covalent Organic Frameworks. *J. Am. Chem. Soc.* **140**, 5138-5145, doi:10.1021/jacs.7b13558 (2018).

6 Cao, L. *et al.* Weakly Humidity-Dependent Proton-Conducting COF Membranes. *Adv. Mater.* **32**, 2005565, doi:10.1002/adma.202005565 (2020).

7 Adamski, M. *et al.* Highly Stable, Low Gas Crossover, Proton-Conducting Phenylated Polyphenylenes. *Angew. Chem. Int. Edit.* **56**, 9058-9061, doi:10.1002/anie.201703916 (2017).

8 Miyatake, K., Chikashige, Y., Higuchi, E. & Watanabe, M. Tuned Polymer Electrolyte Membranes Based on Aromatic Polyethers for Fuel Cell Applications. *J. Am. Chem. Soc.* **129**, 3879-3887, doi:10.1021/ja0672526 (2007).

9 Li, N., Lee, S. Y., Liu, Y.-L., Lee, Y. M. & Guiver, M. D. A new class of highly-conducting polymer electrolyte membranes: Aromatic ABA triblock copolymers. *Energy Environ. Sci.* **5**, 5346-5355, doi:10.1039/C1EE02556B (2012).

10 Roy, A., Yu, X., Dunn, S. & McGrath, J. E. Influence of microstructure and chemical composition on proton exchange membrane properties of sulfonated–fluorinated, hydrophilic–hydrophobic multiblock copolymers. *J. Membr. Sci.* **327**, 118-124, doi:10.1016/j.memsci.2008.11.016 (2009).

11 Takamuku, S. & Jannasch, P. Multiblock Copolymers with Highly Sulfonated Blocks Containing Di- and Tetrasulfonated Arylene Sulfone Segments for Proton Exchange Membrane Fuel Cell Applications. *Adv. Energy Mater.* **2**, 129-140, doi:10.1002/aenm.201100515 (2012).

12 Kang, N. R., Pham, T. H. & Jannasch, P. Polyaromatic Perfluorophenylsulfonic Acids with High Radical Resistance and Proton Conductivity. *ACS Macro Lett.* **8**, 1247-1251, doi:10.1021/acsmacrolett.9b00615 (2019).

13 Chandra, S. *et al.* Interplaying Intrinsic and Extrinsic Proton Conductivities in Covalent Organic Frameworks. *Chem. Mat.* **28**, 1489-1494, doi:10.1021/acs.chemmater.5b04947 (2016).

14 Shinde, D. B. *et al.* A mechanochemically synthesized covalent organic framework as a proton-conducting solid electrolyte. *J. Mater. Chem. A* **4**, 2682-2690, doi:10.1039/C5TA10521H (2016).

15 Montoro, C. *et al.* Ionic Conductivity and Potential Application for Fuel Cell of a Modified Imine-Based Covalent Organic Framework. *J. Am. Chem. Soc.* **139**, 10079-10086, doi:10.1021/jacs.7b05182 (2017).

16 Ma, H. *et al.* Cationic Covalent Organic Frameworks: A Simple Platform of Anionic Exchange for Porosity Tuning and Proton Conduction. *J. Am. Chem. Soc.* **138**, 5897-5903, doi:10.1021/jacs.5b13490 (2016).

17 Xu, H., Tao, S. & Jiang, D. Proton conduction in crystalline and porous covalent organic frameworks. *Nat. Mater.* **15**, 722-726, doi:10.1038/nmat4611 (2016).

18 Chandra, S. *et al.* Phosphoric Acid Loaded Azo (−N=N−) Based Covalent Organic Framework for Proton Conduction. *J. Am. Chem. Soc.* **136**, 6570-6573, doi:10.1021/ja502212v (2014).

19 Sasmal, H. S. *et al.* Superprotonic Conductivity in Flexible Porous Covalent Organic Framework Membranes. *Angew. Chem. Int. Edit.* **57**, 10894-10898, doi:10.1002/anie.201804753 (2018).

20 Peng, Y. *et al.* Mechanoassisted Synthesis of Sulfonated Covalent Organic Frameworks with High Intrinsic Proton Conductivity. *ACS Appl. Mater. Interfaces* **8**, 18505-18512, doi:10.1021/acsami.6b06189 (2016).

21 Yang, Y. *et al.* Combined Intrinsic and Extrinsic Proton Conduction in Robust Covalent Organic Frameworks for Hydrogen Fuel Cell Applications. *Angew. Chem. Int. Edit.* **59**, 3678-3684, doi:10.1002/anie.201913802 (2020).

22 Tao, S. *et al.* Confining H3PO4 network in covalent organic frameworks enables proton super flow. *Nat. Commun.* **11**, 1981, doi:10.1038/s41467-020-15918-1 (2020).

23 Li, S. *et al.* Enhanced Proton Conductivity of Imidazole-Doped Thiophene-Based Covalent Organic Frameworks via Subtle Hydrogen Bonding Modulation. *ACS Appl. Mater. Interfaces* **12**, 22910-22916, doi:10.1021/acsami.0c04002 (2020).

24 Ranjeesh, K. C. *et al.* Imidazole-Linked Crystalline Two-Dimensional Polymer with Ultrahigh Proton-Conductivity. *J. Am. Chem. Soc.* **141**, 14950-14954, doi:10.1021/jacs.9b06080 (2019).

25 Meng, Z., Aykanat, A. & Mirica, K. A. Proton Conduction in 2D Aza-Fused Covalent Organic Frameworks. *Chemistry of Materials* **31**, 819-825, doi:10.1021/acs.chemmater.8b03897 (2019).

26 Chen, S. *et al.* Tuning proton dissociation energy in proton carrier doped 2D covalent organic frameworks for anhydrous proton conduction at elevated temperature. *J. Mater. Chem. A* **8**, 13702-13709, doi:10.1039/D0TA04488A (2020).

27 Wu, X. *et al.* Perfluoroalkyl-functionalized Covalent Organic Frameworks with Superhydrophobicity for Anhydrous Proton Conduction. *J. Am. Chem. Soc.* **142**, 14357-14364, doi:10.1021/jacs.0c06474 (2020).

28 Liu, L. *et al.* Surface-Mediated Construction of Ultrathin Free-standing Covalent Organic Framework Membrane for Efficient Proton Conduction. *Angew. Chem. Int. Edit.* **60**, 14875-14880, doi:10.1002/anie.202104106 (2021).

29 Fan, C. *et al.* Scalable fabrication of crystalline COF membrane from amorphous polymeric membrane. *Angew. Chem. Int. Edit.* **60**, 18051-18058, doi:10.1002/anie.202102965 (2021).

30 Shin, D. W. *et al.* Sulfonated Poly(arylene sulfide sulfone nitrile) Multiblock Copolymers with Ordered Morphology for Proton Exchange Membranes. *Macromolecules* **46**, 7797-7804, doi:10.1021/ma400889t (2013).

31 Miyake, J. *et al.* Design of flexible polyphenylene proton-conducting membrane for next-generation fuel cells. *Sci. Adv.* **3**, eaao0476, doi:10.1126/sciadv.aao0476 (2017).

32 Matsumoto, K., Higashihara, T. & Ueda, M. Star-Shaped Sulfonated Block Copoly(ether ketone)s as Proton Exchange Membranes. *Macromolecules* **41**, 7560-7565, doi:10.1021/ma8015163 (2008).

33 Long, Z., Miyake, J. & Miyatake, K. Proton exchange membranes containing densely sulfonated quinquephenylene groups for high performance and durable fuel cells. *J. Mater. Chem. A* **8**, 12134-12140, doi:10.1039/d0ta03435e (2020).

34 Tang, H., Geng, K., Hu, Y. & Li, N. Synthesis and properties of phosphonated polysulfones for durable high-temperature proton exchange membranes fuel cell. *J. Membr. Sci.* **605**, doi:10.1016/j.memsci.2020.118107 (2020).
